# User Experience of a Virtual Reality–Based Treadmill for Children With a Chronic Disease Affecting Physical Health: Cross-Sectional Feasibility Study

**DOI:** 10.2196/82953

**Published:** 2026-04-29

**Authors:** Capucine Hennequin, Lena Carcreff, Adélie Christiaens, Mickaël Dinomais, Josselin Demas

**Affiliations:** 1 Faculty of Medicine University of Rouen Normandy Rouen France; 2 Rouen University Hospital Rouen France; 3 Department of Physical and Rehabilitation Medicine Angers University Hospital Angers France; 4 Angers Living Lab in Hospital Geriatrics Department of Geriatric Medicine and Rehabilitation Angers University Hospital Angers France; 5 Angevin Research Laboratory for Systems Engineering, EA7315 University of Angers Angers France; 6 Training Institutes Laval Hospital Center Laval France

**Keywords:** cerebral palsy, neuromuscular diseases, obesity, pediatrics, walking, rehabilitation, virtual reality, user-centered design

## Abstract

**Background:**

For children with chronic conditions affecting physical health and who require long-term care, the use of a connected treadmill for gait training as part of a home program can be a way to promote motivation in rehabilitation. Furthermore, the device must be evaluated by all user groups to ensure that its development best meets the rehabilitation needs of children.

**Objective:**

The study aimed to assess the user experience of a connected treadmill called *Amy*—with both immersive and nonimmersive virtual reality—among children with a chronic disease impacting physical health, as well as their parents and therapists, to explore the feasibility and potential of such a device for home-based rehabilitation in this population.

**Methods:**

Children with cerebral palsy, neuromuscular diseases, or obesity, along with one of their parents and rehabilitation therapists, were recruited. The study involved evaluating preexisting *Amy* solutions and collecting user experience feedback from participants with questionnaires. *Amy* solutions consisted of immersive virtual reality (using a virtual reality headset) and nonimmersive (tablet-based) games, both controlled through body movements on a treadmill conceived to train walking and balance. Questionnaires were the short version of the User Experience Questionnaire; the Usability Metric for User Experience; the Virtual Reality Sickness Questionnaire; a customized questionnaire evaluating comfort, fun, sense of presence and immersion; and a customized questionnaire evaluating parent’s perception.

**Results:**

Twenty-eight children, 28 parents, and 18 therapists participated in the study. Compared with User Experience Questionnaire benchmark data, the overall results with immersive and nonimmersive virtual reality in all participants were in the range of 10% best results or in the “excellent” category. The mean (95% CI) scores for each group of participants, with nonimmersive and immersive virtual reality, were as follows: 1.9 (1.6-2.2) and 2.1 (1.6-2.5) for children, 2.0 (1.7-2.2) and 2.3 (2.0-2.5) for parents, and 1.4 (1.1-1.7) and 1.4 (1.1-1.7) for therapists, respectively. User experience was significantly better for children and parents than for therapists (*P* adjusted .001). From the Usability Metric for User Experience, participants rated the *Amy* treadmill’s usability as “good to excellent” on the System Usability Scale, regardless of whether immersive virtual reality was used. Immersive virtual reality was well tolerated by children. Children experienced immersive virtual reality positively in terms of comfort, immersion, presence, and fun. Parents’ acceptability of the connected treadmill was positively assessed.

**Conclusions:**

This study is, to our knowledge, the first to assess the user experience of a playful treadmill-based virtual environment controller in children with chronic conditions affecting physical health in a user-centered and multidisciplinary team-based approach. This initial test demonstrates promising potential for using the connected treadmill as a rehabilitation tool. Therapists may need improvements to better meet their expectations, highlighting the importance of further iterations to align technological features and practical clinical context.

## Introduction

Chronic diseases in children are a major public health issue. Over the past few years, their prevalence has increased, with 10%-30% of children affected by a chronic disease [1]. Cerebral palsy, neuromuscular diseases, and obesity are chronic conditions characterized by physical impairment, particularly affecting the musculoskeletal and cardiorespiratory systems [2-4]. This physical impairment affects the gross motor function and walking ability of these children. Indeed, the walking endurance is reduced in children with cerebral palsy compared with their typically developing peers, even among those with the highest functional abilities [5]. The walking endurance is also reduced in children with obesity or neuromuscular diseases [6,7]. Combined with personal and environmental factors, this physical impairment can therefore lead to activity limitations and participation restrictions, affecting the child’s quality of life [2,4,6,8].

Functional gait training, including treadmill training, consists in endurance, balance, and strength training exercises. In children with cerebral palsy, neuromuscular diseases, or obesity, it can improve gross motor function, balance, and walking ability, as well as metabolic, cardiovascular, and respiratory parameters [9-14]. Gait training including treadmill training has shown promising results for physical health and functional capacities in children with chronic diseases [13-15]. Although there is no consensus due to the great heterogeneity in terms of duration, frequency, and intensity, it appears that longer programs, conducted over several weeks and with progressive intensity, are preferred [9,10,12-14,16]. Future research is required to recommend the best programs in terms of intensity and duration [11].

Rehabilitation is a huge part of clinical management in those 3 chronic diseases. Thus, maintaining motivation over a long time is a major objective. New technologies and rehabilitation methods, such as the combination of a treadmill with virtual reality, included in a personalized home-based program, could be an ideal way to promote motivation in rehabilitation [10,11]. Home-based programs are considered a useful and feasible complement to therapies in rehabilitation centers, which are sometimes located far from home [17,18]. They offer an opportunity to increase the intensity and repetition of the tasks being practiced, integrating them into children's familiar environment, while still providing a structured framework under the supervision of a therapist with regular feedback and have shown positive effects in improving motor function, cardiometabolic health, and self-care [11,12,19,20]. Virtual reality offers a real-time multisensory experience, promotes motor learning through task repetition and feedback, and enables accurate and personalized performance monitoring, even remotely, thanks to digital devices. Also, virtual reality may enhance enjoyment for children, increase their engagement in physical activity, and improve adherence to rehabilitation programs [9,10,19,21-23]. Nevertheless, the wide variability in exercise modalities, technological supports, and modes of patient participation means that no clear guidelines are currently available to inform the design of new programs [18].

New technologies such as treadmills and virtual reality were initially developed for adults and must be adapted for children, especially for children with disabilities. *Amy* is a device created by the company EzyGain for adults but that can also be adjusted for children. *Amy* is a connected treadmill, meaning that the treadmill itself, through its use, enables interaction with a virtual environment to which it is linked. It was developed as a rehabilitation tool to train walking and balance at home, in an entertaining way, within a telerehabilitation model that includes regular monitoring by a therapist.

Before trying a treadmill equipped with virtual reality and integrating it in a home-based program, we must identify key priorities to assess the device to the target population. The user experience (UX) refers to a person’s interaction with a system. UX is subjective and encompasses 2 main categories [24]. The first category focuses on the pragmatic and functional aspects of the system. The second category focuses on the emotional, hedonic, and aesthetic aspects of the system. These aspects will influence the user’s emotional reactions, resulting in acceptance, positive or negative evaluation of the system, and the intention to use it. A positive UX is therefore essential for prolonged use, especially in future projects involving a home-based rehabilitation program lasting several weeks. UX involves not only the person actively using the system, here the child using the connected treadmill, but also anyone passively observing the user [25]. A multidisciplinary, team-based approach that involves parents and rehabilitation therapists, as well as children, provides the necessary perspectives for user-centered design [20,26]. Parental opinion is a crucial element in the success of an intervention, especially in a home-based program where they are directly involved [20,27]. The therapists’ opinion is also essential. They will need to integrate the device into their therapeutic arsenal and clinical practice, conduct the remote rehabilitation program, personalize it for each child, and educate and coach parents [15,16,22]. Involving all end users from the earliest stages of development allows to work toward finding the best fit between a technological system—in this case, the connected treadmill—and the expectations of children, parents, and therapists, ultimately supporting optimal rehabilitation outcomes [28].

In the field of pediatric rehabilitation, existing literature mainly includes papers focusing on the UX of devices designed for cognitive [29-33] or upper limb rehabilitation [34,35]. To our knowledge, and to date, few technological devices targeting gait training in children have been codeveloped using a UX-driven approach. Ammann-Reiffer et al [23] studied the UX of a virtual reality headset as a rehabilitation tool for everyday walking activities. No study has explored the UX of a treadmill, combined with VR—that is, designed as a game controller—in children with chronic diseases affecting physical health in a user-centered design and a multidisciplinary, team-based approach.

The primary objective of this study was to investigate the UX of a treadmill connected to immersive and nonimmersive virtual reality among children with a chronic disease affecting physical health, as well as their parents and therapists. The secondary objectives were to (1) evaluate the usability of a connected treadmill among children, parents, and therapists; (2) evaluate cybersickness induced by immersive virtual reality in children; (3) evaluate the comfort, fun, sense of presence, and immersion experienced with immersive virtual reality in children; (4) evaluate the parent’s perception of a connected treadmill; and (5) compare UX across the different groups as well as between immersive and nonimmersive virtual reality. We hypothesized that UX would be generally positive among the sample participants, with usability expected as an area for improvement. We also expected virtual reality to be well tolerated, and that comfort, fun, presence, and immersion would receive positive evaluations in immersive mode. Additionally, we anticipated parental heterogenous acceptance of the system. Finally, we hypothesized that UX would be similar across the 3 groups but superior in immersive mode.

## Methods

### Study Design

A cross-sectional feasibility UX study was conducted at the Capucins Rehabilitation Centre and the University Hospital in Angers, France, in October and November 2024. During this initial user test, quantitative data were collected via questionnaires and qualitative data via focus groups. The subject of this paper focuses on quantitative data. The study adheres to the RATE-XR guideline recommended in early-stage evaluations of extended reality applications [36].

### Ethical Considerations

The study was approved by the Angers University Hospital Ethics Committee (approval no. 2024-148). It was also declared to the National Commission for Information Technology and Civil Liberties (registration no. 3490000031/559947). An information letter and an opposition form were provided for each participant either in person or via email. The information letters were tailored to specific age groups (those aged 8-10, 11-14, and 15-17 years and adults). Each child gave verbal assent to participate. Informed consent was obtained from parents on behalf of their child and for their own participation, and from all participating therapists. Participation was voluntary without financial compensation, and individuals could withdraw at any time without consequences. All collected data were anonymized before analysis. All necessary measures have been taken to remove any identifying features from the images included in the manuscript. Written consent regarding the evaluator depicted in one of the figures has been obtained and provided to the journal.

### Recruitment

Children with cerebral palsy, neuromuscular diseases, and overweight or obesity, along with one of their parents and therapists, were invited to participate in this study. Recruitment was based on children and parents meeting eligibility criteria and receiving medical follow-up from the pediatric rehabilitation departments of the 2 centers. Therapists in the relevant departments were invited to participate. Three groups were formed based on disease type: the “CP” group (children with cerebral palsy), the “OB” group (children with overweight or obesity), and the “NM” group (children with neuromuscular disease). Children aged 8-18 years who could walk on a treadmill for 5 minutes, with or without support or mobility aids, were included. Parents were included if their child met the study eligibility criteria. Therapists were included if they were accustomed to working with at least one of the study-eligible populations. All participants were required to (1) speak French fluently enough to complete questionnaires, and (2) provide informed consent. Children with uncontrolled epilepsy were excluded. Adults were excluded if deprived of liberty by judicial or administrative decision or if subject to legal protection measures.

### Description of the Virtual Reality–Based Treadmill

*Amy* is a CE-marked (European Conformity) medical device designed for rehabilitation originally created for adults. It consists of a compact treadmill (65×130 cm) with a robust frame, height-adjustable support bars, an antifall harness for safety, and an integrated force plate for capturing weight shifts and controlling games through body movements. The treadmill supports walking speeds ranging from 0.2 to 3 km/h and accommodates users weighing up to 100 kg. A touch-sensitive tablet connects to the treadmill via Bluetooth Low Energy, enabling interaction games and facilitating remote communication with a therapist. Additionally, the *Amy* system is compatible with the Pico 4 virtual reality headset, allowing integration of immersive rehabilitation modules. The height of the support bars, the harness, and the tablet device stand can be adjusted to suit the child's size. The preexisting *Amy* solutions consisted of exergames aimed at training balance and gait for short periods. Exergames were composed of a sequence of mini games, each played one after the other. The progress and completion of each virtual reality game were made possible by the child’s use of the treadmill. See Table 1 for description of the virtual reality games. Games were launched in an application called EzyGain app (version 4.7.3) available on the Apple store at the time of user testing.

**Table 1:** Description of the virtual reality games (nonexhaustive list).

Screenshot^a^	Motor task	Level of immersion	Game objective	Control modality	Game environment	Progression mechanics
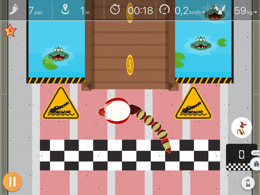	Gaits	Nonimmersive VR^b^	Mediolateral weight transfer while walking	Force plate	Snake-themed	Number of targets and obstacles
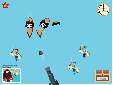	Balance	Nonimmersive VR	Lateral weight shifting	Force plate	Pirate-themed	Number, speed, and size of targets
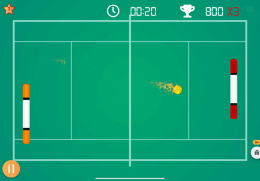	Balance	Nonimmersive VR	Anteroposterior weight shifting	Force plate	Pong-inspired	Target speed
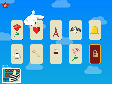	Balance	Nonimmersive VR	Multidirectional weight shifting	Force plate	Memory game	Number and size of cards
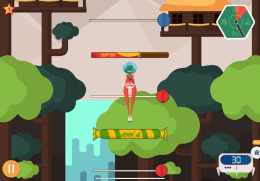	Squat	Nonimmersive VR	Lower limb muscle strengthening	Force plate	Frog-jumping	Height of stages
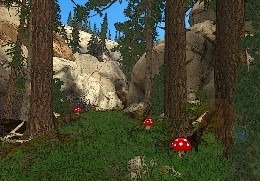	Gait	Immersive VR	Visuomotor target aiming while walking	Speed of the treadmill and VR headset	Forest	Fixed task difficulty
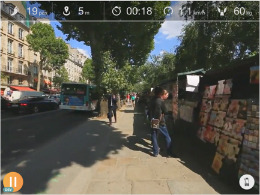	Gait	Immersive VR	Visual exploration while walking	VR headset	Real-world environment (eg, Paris)	Fixed task difficulty

^a^Screenshots provided by EzyGain.

^b^VR: virtual reality.

### Procedure

**Figure 1:**
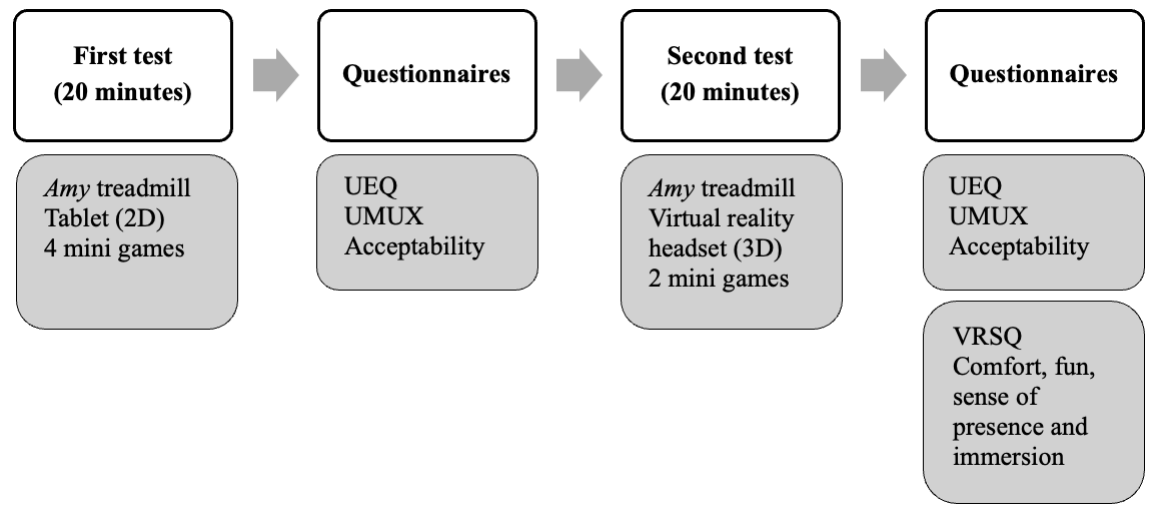
Study participation timeline: testing of a connected treadmill with nonimmersive and immersive virtual reality by children with chronic conditions affecting their physical health and gathering of their user experience as well as that of their parents and therapists. UEQ: User Experience Questionnaire; UMUX: Usability Metric for User Experience; VRSQ: Virtual Reality Sickness Questionnaire.

**Figure 2:**
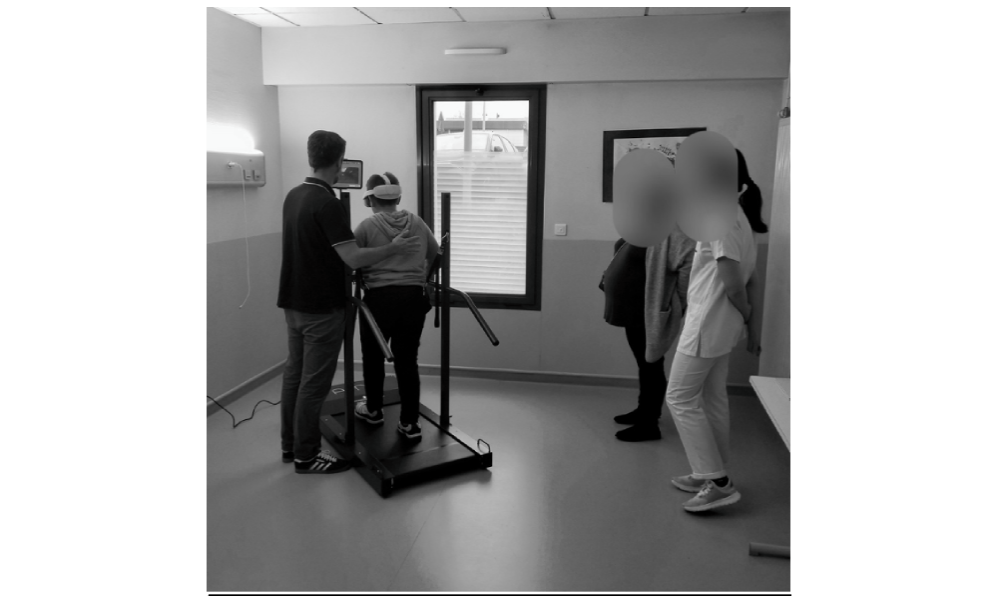
Test session set up. The Amy-connected treadmill being tested by a child with obesity, observed by his parent and a therapist. An investigator is present alongside the child to supervise the session.

The study involved evaluating preexisting *Amy* solutions and collecting UX feedback from participants (Figure 1). Each child participated in 2 consecutive 20-minute sessions the same day, accompanied by one of their parents and a therapist. Each child was first assisted in putting on the safety harness to ensure secure use of the treadmill. Once properly equipped, they stepped onto the treadmill and tried out different games. Between games, the treadmill was stopped so that the child could rest, if necessary. During the test sessions, parents and therapists were purely observers (Figure 2). An investigator was present in the room to supervise, in particular, to help the child settle in and start playing games. In the first session, children used the *Amy* treadmill combined with the tablet-based interface, experiencing 4 nonimmersive (2D) virtual reality games (see examples in Table 1). After completing this session, all participants filled in a set of UX questionnaires. In the second session, children used the *Amy* treadmill with the virtual reality headset, experiencing 2 immersive (3D) virtual reality games (Table 1). Following this session, participants again completed a set of UX questionnaires. While the specific games differed between participants, all children were exposed to a range of game types addressing various motor objectives, as summarized in Table 1.

To minimize the Hawthorne effect and social desirability bias, participants were explicitly informed that the investigators’ role was limited to data collection and that responses should reflect their personal perceptions and experiences. An investigator was present to support the children during questionnaire completion, providing clarification when necessary, given that the questionnaires were not yet validated for this young population. Throughout the entire process, participants provided informal verbal feedback and comments, which contributed to enriching the discussion of this study.

### UX Assessment

Ease with technology was evaluated by a single question: “On a scale of 1 to 10, how comfortable would you say you are with technology?” This single question provided a rough estimate of participants’ level of acceptance of technologies of all kinds.

UX was evaluated using the French version of the User Experience Questionnaire (UEQ) [37]. The UEQ allows for a quick assessment of UX. The short version of the UEQ was used, which consists of 8 items grouped into 2 subscales: pragmatic quality and hedonic quality, each with 4 items. Pragmatic quality assesses efficiency, perspicuity, and dependability; hedonic quality assesses stimulation and originality. Each item is formatted as a Likert scale semantic differential that represents opposites (eg, “simple-complicated”) [37,38]. The range rates between –3 and 3. All participants completed the UEQ’s short version. The short version of the UEQ has been scientifically validated for adults in English (Cronbach a values of 0.85 for the pragmatic quality and 0.81 for the hedonic quality) [37].

Usability was evaluated using the French version of the Usability Metric for User Experience (UMUX). The UMUX is a questionnaire of 4 items evaluating usability components: effectiveness, satisfaction, overall, and efficiency. Each item is a 7-point Likert scale (from 1 “strongly disagree” to 7 “strongly agree”). After being collected, data were converted in a range of 0-100 UMUX score following the author’s guidelines [39]. The advantage of the UMUX is that it is highly correlated with the System Usability Scale (SUS), which is the most widely used usability questionnaire. The UMUX is shorter than the SUS and aligns more closely with the ISO (International Organization for Standardization) 9241-11 (1998) definition of usability and evaluates more specifically the usefulness of a system in addition to usability, unlike the SUS and the UEQ-S [39,40]. All participants completed the UMUX. The UMUX has been scientifically validated for adults in English (Cronbach a value of 0.94) [39].

Cybersickness was evaluated using the Virtual Reality Sickness Questionnaire (VRSQ). It measures motion sickness-like sensations resulting from the use of virtual reality headsets. Only the children completed the VRSQ. They assigned a severity score from 0 to 3 (none, mild, moderate, and severe) to each of the 9 VRSQ items. The 9 items are grouped into 2 subscales: oculomotor discomfort and disorientation. The total score reflects the average of the oculomotor discomfort and disorientation scores out of 100 [41]. The VRSQ has been scientifically validated for adults in English (Cronbach ⍺ values of 0.847 for the oculomotor component and 0.886 for the disorientation component) [41].

A customized questionnaire covering the aspects of comfort, fun, sense of presence, and immersion in the virtual environment with the virtual reality headset was proposed. This questionnaire was based on the study by Ammann-Reiffer et al [23]. Only the children completed the questionnaire. The questions were as follows: “How comfortable did you feel during the game?” “How much did you enjoy performing the task?” “How present did you feel in the virtual environment?” “How much of a problem was it for you not to be able to see the real environment and your body?” Children answered on a visual analogue scale from 1 to 10.

Acceptability from the parent’s point of view was assessed by a customized questionnaire also inspired by the study by Ammann-Reiffer et al [23]. The items were as follows: “The child’s movement on the *Amy* treadmill was not impaired compared with his usual walking,” “The child required no more support than usual,” “The child required no external motivation to walk,” “The child’s involvement was greater than usual (at home/in the usual physiotherapy session),” “The child remained interested and did not seem bored,” and “The use of the treadmill could be a good complement to conventional physiotherapy approaches for this child.” Parents answered on a 7-point Likert scale (from 1 “strongly disagree” to 7 “strongly agree”).

### Outcomes

The primary outcome was the mean scores on the UEQ-S overall and in the pragmatic and hedonic scales. The secondary outcomes were (1) the UMUX mean scores; (2) the VRSQ median scores; (3) responses to the customized questionnaire evaluating sensations of comfort, fun, sense of presence, and immersion; (4) responses to the customized questionnaire evaluating parent’s perception; and (5) comparisons between the different groups of patients and between nonimmersive and immersive virtual reality.

### Sample Size

The sampling strategy was nonprobabilistic and purposive [42]; that is, participants were recruited according to specific criteria, using maximum variation sampling [43] to ensure diversity among participants and, consequently, a wide range of perspectives within the group. The following variability criteria were considered: (1) age of the children and therapists, (2) sex of the children (including both girls and boys in each group), and (3) variety of pathological conditions. The median number of participants in UX studies reported in the literature is 20 [44]. Given the diversity among participants, we expected to include 30 children and consequently 30 parents. We also aimed to include 15 therapists for a total of 75 participants.

### Statistical Analysis

Scores of the standardized scales (UEQ, UMUX, and VRSQ) were calculated as recommended by the reference methods [37,39,41,45]. Descriptive statistics reporting the mean and 95% CI were used to present UEQ-S and UMUX results. Values obtained on the UEQ below –0.8 correspond to a negative assessment, between –0.8 and 0.8 to a neutral assessment, and above 0.8 to a positive assessment [46]. Mean UEQ scores were compared with published reference values using a benchmark dataset created by the author, which contains evaluations of 452 products’ evaluation and 20191 responses [46]. This enabled the evaluated product to be placed in one of 5 categories: “excellent” (in the range of the 10% best results), “good” (10% of the results in the benchmark dataset are better and 75% of the results are worse), “above average” (25% of the results in the benchmark are better than the result for the evaluated product and 50% of the results are worse), “below average” (50% of the results in the benchmark are better than the result for the evaluated product and 25% of the results are worse), and “bad” (in the range of the 25% worst results) [45]. UMUX scores were used to position the usability of the solution on the SUS interpretation scale (“worst imaginable,” “poor,” “acceptable,” “good,” “excellent,” or “best imaginable”) [47]. Descriptive statistics reporting the median and quartiles were used to present results from the VRSQ and customized questionnaires (comfort with technology; comfort, fun, sense of presence, and immersion; and parents’ perception). These scores were calculated for each version of the product tested, that is, with nonimmersive (2D) and immersive (3D) virtual reality.

To compare between-group differences in mean or median scores, we used nonparametric statistical tests, given the low number of participants and the data distribution. Wilcoxon test was used for paired samples. Kruskal-Wallis test was used to compare more than 2 samples. *P* value of less than .05 was considered statistically significant. Bonferroni correction was used to adjust the *P* value in the case of multiple statistical tests.

## Results

### Demographics

Twenty-eight children (13 females; 15 males; mean age 11.9, SD 2.7 years), 28 parents, and 18 therapists participated in the study. Eleven children had cerebral palsy, 9 had obesity, and 8 had neuromuscular disease. Missing data were observed for secondary descriptive variables only (mobility aid and school level). No formal test for missing completely at random was performed. Demographics for the participants are shown in Table 2.

**Table 2:** Descriptive characteristics of the study participants (N=74) presented to provide context for the user experience data collected from children (n=28, part A) with chronic conditions trying a connected treadmill, as well as their parents (n=28, part B) and therapists (n=18, part C).

Characteristics				Participants		
**Part A: children** **(n=28)**						
	**Diagnosis, n (%)**					
		Cerebral palsy	11 (39)			
		Obesity	9 (32)			
		Neuromuscular diseases	8 (29)			
	**Age (years), mean (SD)**					
		Overall	11.9 (2.7)			
		Cerebral palsy	11.3 (2.9)			
		Obesity	13 (2.7)			
		Neuromuscular diseases	11.5 (2.1)			
	**Gender, n (%)**					
		Women	13 (46)			
		Men	15 (54)			
	**School level, n (%)**					
		Elementary school	8 (28)			
		Secondary school	12 (43)			
		High school	3 (11)			
		Medicoeducational institute	1 (3.5)			
		Vocational training	1 (3.5)			
		Missing values	3 (11)			
	**Mobility aid on short distances, n (%)**					
		None	20 (71)			
		Ankle-foot orthosis	5 (18)			
		Crutches	1 (4)			
		Walker	2 (7)			
		Missing values	1 (4)			
	**Ease with technology on a scale of 1-10, median (Q1^a^-Q3^b^)**					
		Overall	8 (6-9)			
**Part B: parents** **(n=28)**						
	**Age (years), mean (SD)**					
		Overall	44.3 (7.5)			
	**Gender, n (%)**					
		Women	21 (75)			
		Men	7 (25)			
	**Ease with technology on a scale of 1-10, median (Q1-Q3)**					
		Overall	7 (7-8)			
**Part C: therapists (n=18)**						
	**Age (years), mean (SD)**					
		Overall	31.6 (11.9)			
	**Gender, n (%)**					
		Women	15 (83)			
		Men	3 (17)			
	**Profession, n (%)**					
		Physiotherapist	5 (28)			
		Adapted physical activity teacher	4 (22)			
		Occupational therapist	8 (44)			
		Medical doctor	1 (6)			
	**Ease with technology on a scale of 1-10, median (Q1-Q3)**					
		Overall	7 (5-7)			

^a^Q1: first quartile.

^b^Q3: third quartile.

### User Experience

**Figure 3:**
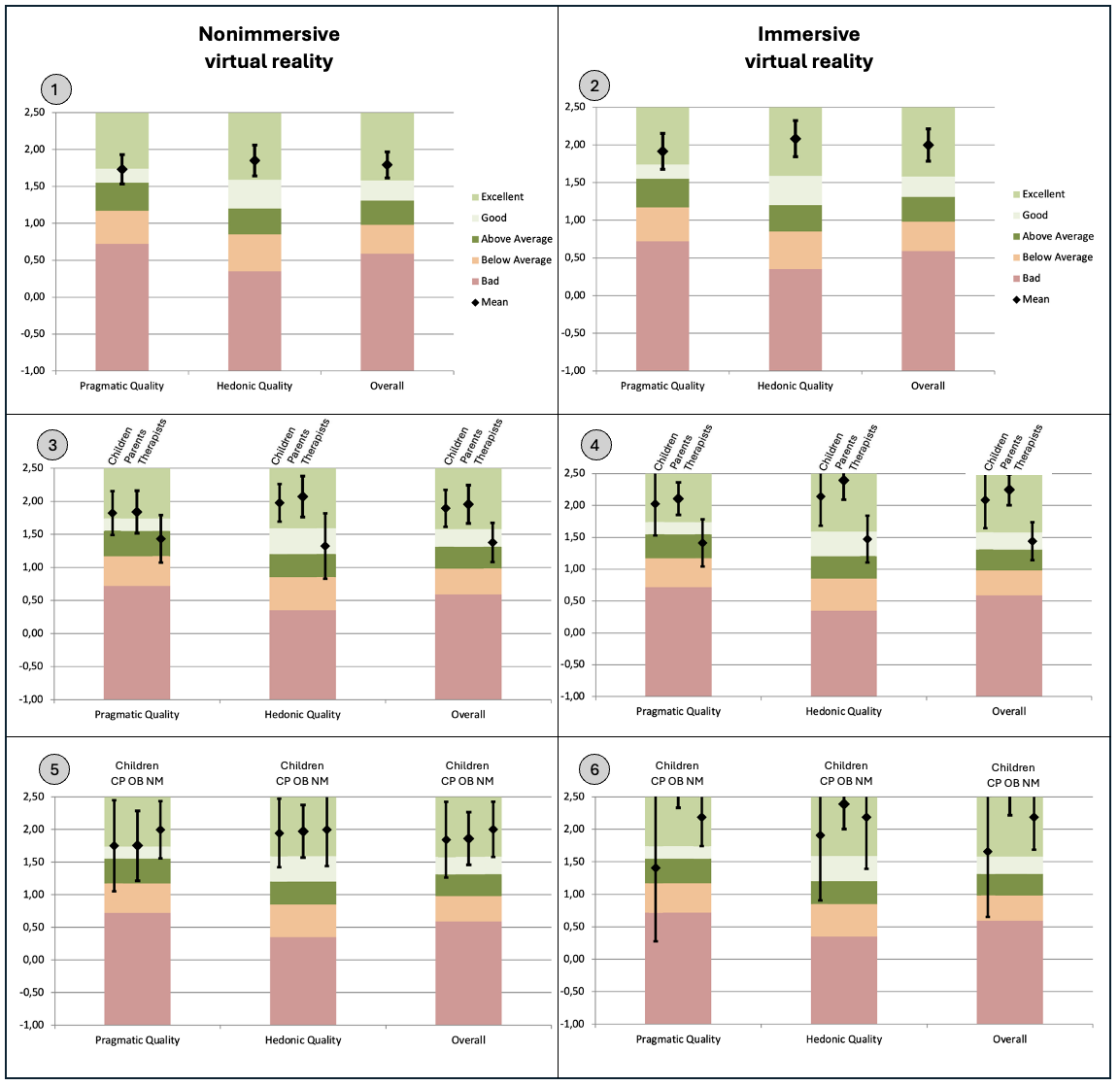
Mean scores and 95% CI bars for the entire short version of the User Experience Questionnaire, pragmatic, and hedonic quality scales, relative to the benchmark. The device evaluated is the Amy treadmill, which incorporates both nonimmersive (left panels) and immersive (right panels) virtual reality. It was tested by children with chronic conditions. User experience data were collected from children, their parents, and therapists. Benchmark graphs come from the Excel tool created by the author [45]. (1) For all participants (N=74), when using nonimmersive virtual reality; (2) for all participants (N=74), when using immersive virtual reality; (3) for children (n=28), parents (n=28), and therapists (n=18), when using nonimmersive virtual reality; (4) for children (n=28), parents (n=28), and therapists (n=18), when using immersive virtual reality; (5) for children, in CP (n=11), OB (n=9), and NM (n=8) groups, when using nonimmersive virtual reality; and (6) for children, in CP (n=11), OB (n=9), and NM (n=8) groups, when using immersive virtual reality. The scale ranges from –3 (fully agree with the negative item) to +3 (fully agree with the positive item). The colored bars represent the ranges for the scale’s mean values. CP: cerebral palsy; NM: neuromuscular; OB: obesity.

The scores to the UEQ-S were all positive, mean values beyond 0.8 representing a positive evaluation according to the author [46]. For all participants, the scores for each scale, with nonimmersive and immersive virtual reality, were as follows: mean 1.8 (95% CI1.6-2.0) and mean 2.0 (95% CI 1.8-2.2) for the overall UEQ-S, mean 1.7 (95% CI 1.5-1.9) and mean 1.9 (95% CI 1.7-2.2) for the pragmatic quality scale, and mean 1.9 (95% CI 1.6-2.1) and mean 2.1 (95% CI 1.8-2.3) for the hedonic quality scale, respectively. The overall UEQ-S, as well as the pragmatic and hedonic quality scales, were also positively evaluated in both immersive and nonimmersive virtual reality separately in children, parents, and therapists, and in children’s subgroups CP, NM, and OB. Mean (SD) UEQ-S responses are shown in Table S1 in Multimedia Appendix 1. Compared with benchmark data, the overall scale means with immersive and nonimmersive virtual reality in all participants were in the range of 10% best results or in the “excellent” category (Figure 3). For all participants, there was a statistically significant difference in favor of immersive virtual reality for the overall UEQ-S (*P*<.001), as well as in the pragmatic (*P*=.001) and hedonic quality scales (*P*<.001). There was a significant difference in favor of immersive virtual reality for children (*P*=.001) and parents (*P*<.001) but not for therapists (*P*=.41).

The overall UEQ-S was significantly better for children and parents than for therapists (*P* adjusted <.001). No difference was found between children and parents (*P* adjusted >.99). Compared with existing values from the benchmark dataset, the—overall, pragmatic, and hedonic quality—scale means with immersive and nonimmersive virtual reality in children and parents were in the “excellent” category. On the contrary, for therapists, the pragmatic quality scale means with immersive and nonimmersive virtual reality were in the “above average” category, and the overall and hedonic quality scale means with immersive and nonimmersive virtual reality were in the “good” category.

No statistical difference was found between the children’s subgroups (*P*=.41). A significant difference in favor of immersive virtual reality compared with nonimmersive virtual reality was found in the OB children’s subgroup (*P*=.001), as opposed to the CP group (*P*=.80) and NM group (*P*=.10).

### Usability

**Figure 4:**
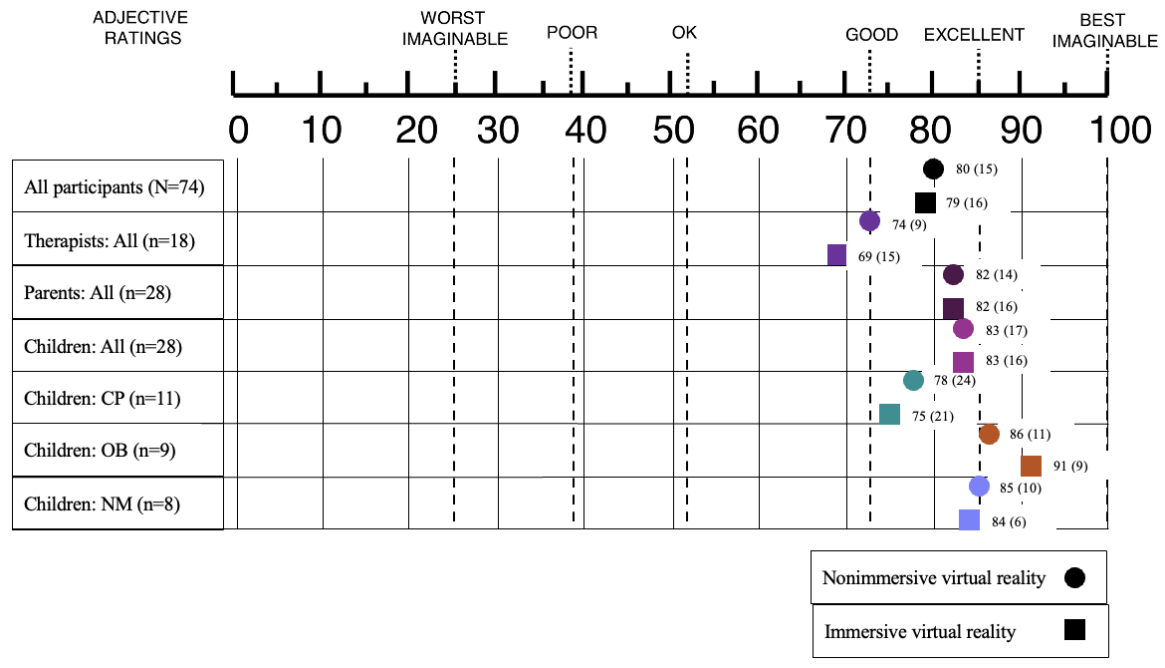
Comparison of Usability Metric for User Experience scores converted to the System Usability Scale normative scale [47]. The score is between 0 and 100. Data are expressed as means (SDs). The device evaluated is the Amy treadmill, which incorporates both nonimmersive (rounds) and immersive (squares) virtual reality. It was tested by children with chronic conditions. Usability data were collected from children, their parents, and therapists. CP: cerebral palsy; NM: neuromuscular: OB: obesity.

Raw UMUX scores are shown in Table S2 in Multimedia Appendix 1. Overall, participants rated the *Amy* treadmill’s usability as “good to excellent” on the SUS scale. Mean (95% CI) scores were 80 (78-82) in 2D and 79 (77-81) in 3D (Figure 4). There was no statistically significant difference between nonimmersive and immersive virtual reality for all participants (*P*=.72).

Children and parents reported similar positive experiences, mean (95% CI) scores being 83 (79-86) and 82 (79-84) in 2D, and 83 (80-86) and 82 (79-85) in 3D, respectively, that is “good to excellent” on the SUS scale. There was no statistically significant difference between nonimmersive and immersive virtual reality for either children (*P*=.47) or parents (*P*=.60), and there was no statistically significant difference between children and parents (*P* adjusted>.99). Therapists rated the system’s usability as “OK to good” on the SUS scale, with mean (95% CI) scores of 74 (72-76) in 2D and 69 (66-73) in 3D, with a significant difference in favor of nonimmersive virtual reality (*P*<.02). The usability was significantly better for children and parents than for therapists (*P* adjusted <.001).

Among children, those with obesity found the system more usable (mean 86, 95% CI 82-90 in 2D; mean 91, 95% CI 88-94 in 3D), followed by those with a neuromuscular disease (mean 85, 95% CI 82-89 in 2D; mean 84, 95% CI 82-86 in 3D) and cerebral palsy (mean 78, 95% CI 71-85 in 2D; mean 75, 95% CI 69-81 in 3D). A significant difference was found only between the OB and CP children’s subgroups (*P* adjusted=.004). A significant difference in favor of immersive virtual reality was found in the OB children’s subgroup (*P*=.005), as opposed to the CP group (*P*=.50) and NM group (*P*=.30).

### Cybersickness

Immersive virtual reality was well tolerated by children, apart from 1 participant in the cerebral palsy group. Overall, symptoms of cybersickness were minimal with a median total score on a 0-100 scale of 4 [3-12]. When present, these symptoms primarily involved oculomotor discomfort rather than disorientation, as indicated by the VRSQ, with a median of 8 (IQR 0-17) for oculomotor discomfort and 0 (IQR 0-7) for disorientation, respectively.

### Comfort, Fun, Sense of Presence, and Immersion

**Figure 5:**
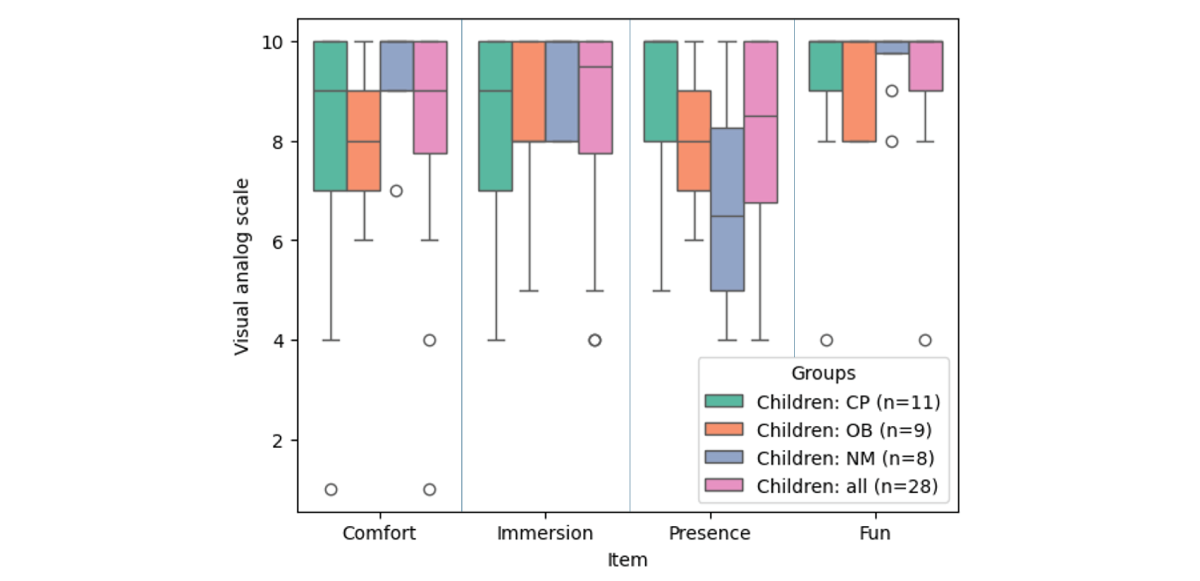
Box plots expressing the scores obtained from the customized questionnaire assessing children’s comfort, immersion, sense of presence, and fun with immersive virtual reality on the Amy treadmill. The scale is a visual analogue scale from 1 to 10. CP: cerebral palsy; NM: neuromuscular; OB: obesity.

Children experienced immersive virtual reality very positively in terms of comfort, immersion, presence, and fun (Figure 5). They felt comfortable during the game (median 9, IQR 8-10). The children reported no difficulty with immersion in the virtual environment (median 10, IQR 8-10). Children reported a strong sense of presence within the virtual environment (median 9, IQR 7-10) and expressed enjoyment while using the treadmill games (median 10, IQR 9-10). Results were similar in children with cerebral palsy, obesity, and neuromuscular disease (*P*=.52).

### Acceptability

**Figure 6:**
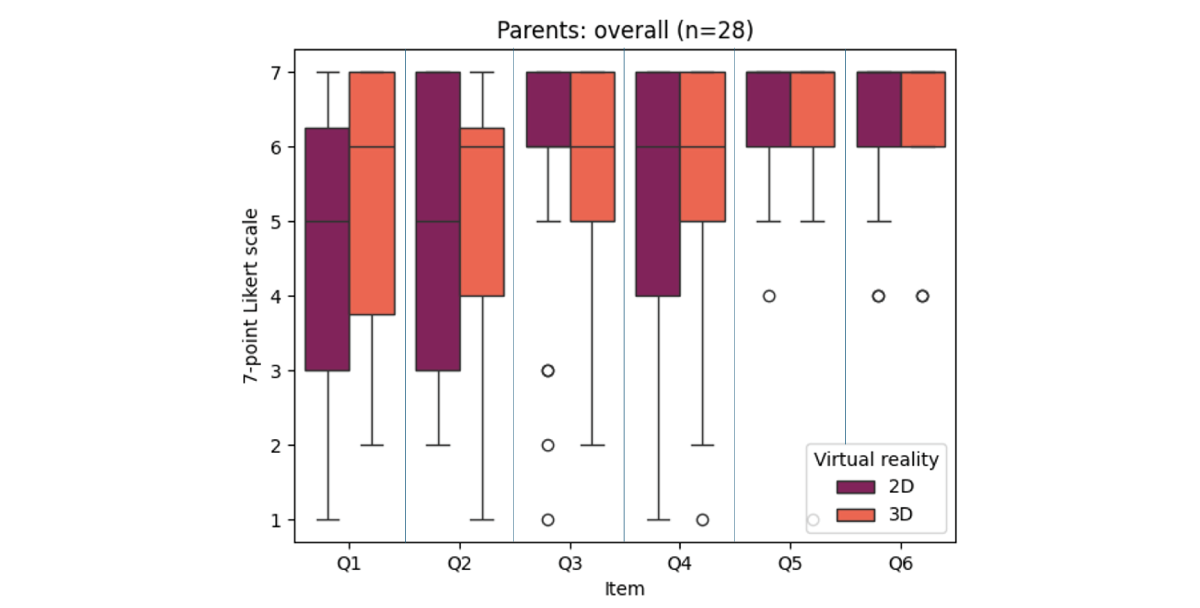
Box plots illustrating the scores obtained for each item in the customized questionnaire measuring parents’ acceptability of their children’s experience with the Amy treadmill under both nonimmersive and immersive virtual reality conditions. Q1: The child’s movement on the Amy treadmill was not impaired compared with his usual walking. Q2: The child required no more support than usual. Q3: The child required no external motivation to walk. Q4: The child’s involvement was greater than usual (at home or in the usual physiotherapy session). Q5: The child remained interested and did not seem bored. Q6: The use of the treadmill could be a good complement to conventional physiotherapy approaches for this child. The scale ranges from 1 (strongly disagree) to 7 (strongly agree). Q: quartile.

Parents’ perception of the connected treadmill was positively assessed (Figure 6). According to the parents, out of 28 children, 8 (28%) exhibited impaired walking on the treadmill when using nonimmersive virtual reality versus 7 (25%) with immersive virtual reality. Parents reported that 8 children needed more support than usual when using nonimmersive virtual reality (8/28, 28%), compared with 5 children with immersive virtual reality (5/28, 18%). Most of the children did not require any external motivation to walk (24/28, 86% with nonimmersive virtual reality and 25/28, 89% with immersive virtual reality). Their engagement seemed increased compared with conventional physiotherapy (24/28, 86% with nonimmersive and immersive virtual reality). Only 1 child seemed bored with immersive virtual reality. All the children remained interested with nonimmersive virtual reality. Apart from a neutral opinion, all parents felt that using the connected treadmill could be a good complement to conventional physiotherapy approaches for their child. Overall, for parents, results were similar between nonimmersive and immersive virtual reality (*P*=.19).

## Discussion

### Principal Findings

In this study, we evaluated the UX of a virtual reality–based treadmill as a rehabilitation tool in a sample of 74 individuals, including children with a chronic disease impacting physical health, their parents, and therapists. Participants reported an excellent UX from testing the connected treadmill about hedonic and pragmatic aspects. The findings showed that the virtual reality–based treadmill presented excellent usability. The immersive virtual reality system was well tolerated in terms of cybersickness; feelings of comfort, fun, sense of presence, and immersion were highly positive. For all participants, UX was better with the immersive virtual reality system than with the nonimmersive virtual reality system. The virtual reality–based treadmill was largely perceived positively by the parents. Children and parents reported greater UX than therapists.

### Interpretation and Comparison With Prior Work

The overall UX evaluation of the virtual reality–based treadmill was excellent, as shown by the results of the UEQ-S. Compared with the benchmark reference data, the results on the connected treadmill were in the top 10%, indicating a product of excellent quality [45]. The pragmatic qualities of the connected treadmill were evaluated positively in terms of dependability, perspicuity, and efficiency. The virtual reality–based treadmill was easy to understand and use, secure, and predictable. This was supported by the results of the UEQ and the UMUX. The UMUX also adds the dimension of usefulness, as the system has been assessed as meeting the children’s needs. The hedonic qualities of the connected treadmill were evaluated positively in terms of stimulation and novelty. Good rates in the UEQ-S were reported on interest, fun, motivation, innovation. Feelings of comfort, immersion, sense of presence, and fun with immersive virtual reality were positively rated by all children. Moreover, the virtual reality headset was well tolerated. Only 1 child, aged 9 years, showed important signs of cybersickness, especially oculomotor discomfort. These results are in line with those reported by Ammann-Reiffer et al [23], who investigated the UX of an immersive virtual reality system to train everyday walking activities in children with a neurologic disease. This virtual reality–based treadmill, designed as a rehabilitation tool for gait and balance training through direct interaction between the treadmill and virtual reality games, generated a positive UX in terms of pragmatic and hedonic qualities, with positive emotions reported in all participants. Control of both immersive and nonimmersive environments is achieved directly through the treadmill, via weight shifts under static and dynamic conditions and adjustments in treadmill speed, providing a convincing UX. Therefore, it has the potential to be adopted by all end users and to motivate children to participate in physical rehabilitation, either more often or for an extended period. As previously showed in the study by Phelan et al [34], who tested immersive virtual reality in children with upper limb injuries, being entertained by virtual reality can increase children’s participation to rehabilitation, as the fun induced by virtual reality keeps them distracted from the difficulties of exercising. This has to be confirmed by a subsequent feasibility study with long-term use of the device in the target environment, that is, at home.

From the parents’ perspective, only a few children exhibited gait disturbances and required additional support (from the bars or the harness), both with nonimmersive and immersive virtual reality. Parents felt that their child was motivated, interested, and engaged during the session, and they considered the use of the connected treadmill to be a valuable complement to conventional rehabilitation methods. The findings align with those of Phelan et al [34], where parents reported a positive UX and showed interest in using the virtual reality system at home. For all participants, there was a statistically significant difference in favor of immersive virtual reality in the UEQ but not in the UMUX questionnaire. The difference could therefore lie in the hedonic aspects that the UEQ investigates unlike the UMUX [40]. According to Winter et al [48], who tested the UX of treadmill training with semi-immersive and immersive virtual reality for patients with gait disorders, better results are explained by the higher sense of presence experienced in the immersive condition compared with the semi-immersive condition.

While the results were positive overall, they were significantly better in children and parents than in therapists, in both the UEQ and the UMUX. Possible explanations could be, on the one hand, functional—relating to the technical aspects of the treadmill—and, on the other hand, associated with the games, such as their design, interactivity, and coordination with the treadmill. Some participants complained about the mismatch between the speed of the games and the speed of the treadmill, which is limited to 3 km/hour. They reported some technical difficulties and simplistic esthetics of the different games. We hypothesize that during this first session, therapists anticipated using the connected treadmill in a more complex situation, that is, at home, under supervision, as part of a rehabilitation program lasting several weeks. As a result, and because of their technical knowledge, they may have had higher expectations of the virtual reality–based treadmill. Given their role as therapists, they may be looking for a device that is safe, reliable, as unobtrusive as possible so that it can be integrated into their workload, and as personalized as possible for patients. The engagement of therapists remains essential for the future implementation of a virtual reality rehabilitation device [28]. In the study by Phelan et al [34], therapists report that immersive virtual reality in children with upper limb injuries can be a good alternative rehabilitation tool and can promote positive interaction between patients and therapists. However, this is subject to several conditions: the system must be customizable to each patient, activities must be varied and progressive, and therapists must be properly trained [34]. Ammann-Reiffer et al [23] reported favorable results in terms of therapists’ acceptability. They found that children were focused and motivated and, above all, were not restricted in their movements by the virtual reality system.

The results were in favor of some difference between the children’s subgroups. Children with obesity showed better usability results than other children, especially with immersive virtual reality. Children with cerebral palsy and neuromuscular diseases were younger and more functionally limited than children with obesity. None of the obese children required a mobility aid. We hypothesize that younger age and greater functional impairment could make it more difficult to use the connected treadmill. Cerebral visual impairment, which is frequently observed among children with cerebral palsy [49], could account for the poorer results observed in this population, although very limited data are available in the literature and further research is warranted.

### Limitations

This study has several limitations. The participant group was heterogeneous, particularly among children, in terms of age and pathology. This enabled us to provide a range of opinions for a UX study but made comparisons difficult and is not intended to be generalized. Regarding UX, and especially usability, part of the virtual reality system was set up by the caregiver. As a result, children did not test the usability of the connected treadmill in its entirety. It could also be interesting to involve parents in the installation of the system in a future context of a home program where one of their roles would be to supervise the treadmill sessions. Ensuring usability is essential, especially for systems intended for long-term use. Two 20-minute sessions UX test were insufficient, and test sessions will need to be repeated throughout the design and improvement process to confirm the trend of these initial results and to project long-term use.

Acceptability from the therapists’ point of view could not be assessed, as it was not possible to match the children with their respective therapists for logistical and organizational reasons. This could be interesting for future studies in order to supplement the results. Although the questionnaires have been translated into French and used in several French language studies and/or with children, they have not yet been formally psychometrically validated in an independently published study. The various items were not specifically adapted to the vocabulary and cognitive abilities of children. Some items of the customized questionnaires were possibly too complex or not clear enough, which may have led to inconsistent answers. This may have been the case in the parents’ perception questionnaire, particularly for the first 2 questions, where a wide variety of responses was observed. Furthermore, the risk of the Hawthorne effect and social desirability bias on participants’ responses to questionnaires cannot be ruled out. Methodologically, and in order to limit the order effect, it could have been valuable to randomize the 2 test sessions. It might have also been interesting to consider motivation for physical activity and the family’s socioeconomic context, as these factors could potentially have an impact on subsequent use of the device in a several-week home rehabilitation program.

### Future Directions

This initial UX session of the connected treadmill, conducted in a supervised environment, demonstrated promising potential within our sample. These findings represent a first step in the iterative codevelopment of the connected treadmill. The device and its UX will be refined based on user feedback to strengthen children’s and parents’ engagement and foster greater acceptance from therapists. Integrating the findings from both quantitative and qualitative studies may enhance accuracy through complementarity. Thus, future studies, quantitative and qualitative, will be necessary to assess UX after the company has developed the games and the treadmill—first in a supervised environment and subsequently in real-life situations in the children’s homes. The objective, from a home program perspective, is to provide a comprehensive program featuring numerous progressive games through an engaging interface, enabling children to participate actively while being supervised by therapists.

### Conclusions

This is the first study to explore the UX of a treadmill designed as a playful game controller of virtual environments, in children with chronic diseases affecting physical health, in a user-centered design and a multidisciplinary, team-based approach. This initial test demonstrates promising potential for using the connected treadmill as a rehabilitation tool. Improvements are required to better meet therapists’ expectations. This underlines the importance of further iterations to achieve the best alignment between technological features and practical clinical context for those children. Consequently, UX will be reassessed after the games and the treadmill have been enhanced, first in a supervised environment and subsequently in real-life settings within the children’s homes, through extended-duration trials.

## Appendix

Multimedia Appendix 1 Detail of the scores obtained for each item of the short version of the User Experience Questionnaire and the Usability Metric for User Experience.

## Abbreviations

CE: European Conformity
CP: cerebral palsy
ISO: International Organization for Standardization
NM: neuromuscular
OB: obesity
SUS: System Usability Scale
UEQ: User Experience Questionnaire
UMUX: Usability Metric for User Experience
UX: user experience
VRSQ: Virtual Reality Sickness Questionnaire

## Data Availability

The datasets generated during this study are available from the corresponding author on reasonable request.
