# Supplementary material for: User Experience of a Virtual Reality–Based Treadmill for Children With a Chronic Disease Affecting Physical Health: Cross-Sectional Feasibility Study
Source: JMIR Serious Games. 2026 Apr 29;14:e82953. doi: 10.2196/82953 (PMC13173068; doi:10.2196/82953)
Supplement: Multimedia Appendix 1 [file games_v14i1e82953_app1.docx]

Multimedia Appendix 1

Table S1. Scores obtained on UEQ-S items and scales for all participants; in children, parents and therapists; and in each subcategory.

In each row, the first row of scores corresponds to results with non-immersive virtual reality, and the second row in bold to results with immersive virtual reality. The scale ranges from -3 (fully agree with the negative item) to +3 (fully agree with the positive item). Data are expressed as means (standard deviations).

| UEQ-S  items | Children | | | | Parents | | | | Therapists | | | | Overall  (N=74) |
| --- | --- | --- | --- | --- | --- | --- | --- | --- | --- | --- | --- | --- | --- |
|  | NM  (n=8) | OB  (n=9) | CP  (n=11) | Overall  (n=28) | NM  (n=8) | OB  (n=9) | CP  (n=11) | Overall  (n=28) | NM  (n=9) | OB  (n=5) | CP  (n=4) | Overall  (n=18) |  |
| Obstructive / Supportive | 0.9 (1.6)  **1.4 (1.6)** | 1.2 (1.6)  **2.3 (0.9)** | 1.4 (1.9)  **1.1 (2.3)** | 1.2 (1.7)  **1.6 (1.8)** | 1.0 (1.1)  **1.6 (1.4)** | 1.1 (1.9)  **2.1 (0.9)** | 1.6 (1.3)  **2.0 (0.9)** | 1.3 (1.4)  **1.9 (1.1)** | 1.2 (0.8)  **1.3 (0.9)** | 1.0 (0.7)  **0.8 (1.9)** | 1.8 (1.3)  **1.5 (1.3)** | 1.3 (0.9)  **1.2 (1.3)** | 1.2 (1.4)  **1.6 (1.4)** |
| Complicated / Easy | 1.9 (1.4)  **2.0 (1.2)** | 1.8 (1.3)  **2.8 (0.4)** | 1.2 (1.6)  **1.1 (2.1)** | 1.6 (1.4)  **1.9 (1.6)** | 1.8 (1.7)  **1.9 (1.2)** | 1.9 (1.5)  **2.3 (0.7)** | 1.6 (0.7)  **2.0 (1.0)** | 1.8 (1.3)  **2.1 (1.0)** | 1.6 (1.0)  **0.8 (1.3)** | 1.2 (1.8)  **2.0 (1.2)** | 1.8 (1.0)  **0.3 (1.5)** | 1.5 (1.2)  **1.0 (1.4)** | 1.6 (1.3)  **1.8 (1.4)** |
| Inefficient / Efficient | 2.5 (0.9)  **2.5 (0.5)** | 2.0 (0.9)  **2.7 (0.5)** | 1.5 (1.9)  **1.5 (2.3)** | 2.0 (1.4)  **2.2 (1.6)** | 2.1 (1.0)  **2.1 (0.8)** | 2.3 (1.1)  **2.6 (0.7)** | 2.1 (0.7)  **1.9 (0.7)** | 2.2 (0.9)  **2.2 (0.8)** | 1.1 (1.4)  **1.4 (0.9)** | 1.4 (1.1)  **1.8 (1.3)** | 2.0 (1.2)  **2.3 (1.0)** | 1.4 (1.2)  **1.7 (1.0)** | 1.9 (1.2)  **2.1 (1.2)** |
| Confusing / Clear | 2.5 (1.1)  **2.9 (0.4)** | 2.0 (1.1)  **2.8 (0.4)** | 2.9 (0.3)  **1.9 (1.8)** | 2.5 (0.9)  **2.5 (1.2)** | 2.3 (0.7)  **2.5 (0.5)** | 1.9 (0.9)  **1.9 (1.9)** | 2.3 (0.9)  **2.4 (0.7)** | 2.1 (0.8)  **2.3 (1.2)** | 1.8 (1.3)  **1.3 (1.3)** | 1.2 (0.8)  **2.2 (0.8)** | 1.5 (0.6)  **2.3 (1.5)** | 1.6 (1.0)  **1.8 (1.3)** | 2.1 (1.0)  **2.2 (1.2)** |
| Pragmatic quality | 1.9 (0.7)  **2.2 (0.6)** | 1.7 (0.8)  **2.6 (0.5)** | 1.8 (1.2)  **1.4 (1.9)** | 1.8 (0.9)  **2.0 (1.3)** | 1.8 (0.9)  **2.1 (0.7)** | | | | 1.4 (0.8)  **1.4 (0.8)** | | | | 1.7 (0.9)  **1.9 (1.0)** |
| Boring / Exciting | 1.8 (1.4)  **1.9 (1.5)** | 2.1 (0.9)  **2.6 (0.7)** | 1.4 (1.8)  **2.0 (1.8)** | 1.7 (1.4)  **2.1 (1.4)** | 2.0 (1.4)  **2.3 (0.9)** | 1.8 (1.2)  **2.8 (0.4)** | 1.9 (0.8)  **1.8 (1.6)** | 1.9 (1.1)  **2.3 (1.2)** | 1.7 (1.3)  **1.0 (1.2)** | 1.0 (1.6)  **0.6 (1.1)** | 1.8 (1.0)  **2.5 (1.0)** | 1.5 (1.3)  **1.2 (1.3)** | 1.7 (1.3)  **2.0 (1.4)** |
| Not interesting / Interesting | 2.5 (0.5)  **2.9 (0.4)** | 2.3 (0.7)  **2.4 (0.7)** | 1.9 (1.3)  **1.8 (1.8)** | 2.2 (1.0)  **2.3 (1.3)** | 2.3 (1.4)  **2.4 (0.9)** | 2.6 (0.7)  **2.8 (0.4)** | 2.0 (0.6)  **2.1 (1.1)** | 2.3 (0.9)  **2.4 (0.9)** | 1.9 (0.9)  **2.0 (0.9)** | 1.6 (1.3)  **1.6 (1.1)** | 1.8 (1.0)  **2.3 (1.0)** | 1.8 (1.0)  **1.9 (1.0)** | 2.1 (1.0)  **2.3 (1.1)** |
| Conventional / Inventive | 2.3 (1.2)  **2.5 (1.4)** | 1.8 (1.0)  **2.7 (0.5)** | 2.6 (0.9)  **2.0 (1.9)** | 2.3 (1.0)  **2.4 (1.4)** | 2.1 (1.0)  **2.5 (0.5)** | 2.0 (1.3)  **2.7 (0.7)** | 2.3 (0.8)  **2.4 (1.0)** | 2.1 (1.0)  **2.5 (0.8)** | 1.0 (1.9)  **1.6 (0.9)** | 1.6 (1.1)  **1.6 (1.1)** | 1.3 (1.5)  **1.3 (1.0)** | 1.2 (1.6)  **1.5 (0.9)** | 2.0 (1.2)  **2.2 (1.2)** |
| Usual / Leading edge | 1.5 (1.1)  **1.5 (1.7)** | 1.7 (1.4)  **1.9 (1.6)** | 1.9 (1.2)  **1.8 (1.8)** | 1.7 (1.2)  **1.8 (1.7)** | 1.9 (1.0)  **2.4 (0.5)** | 2.0 (0.9)  **2.6 (0.7)** | 2.1 (0.9)  **2.4 (0.9)** | 2.0 (0.9)  **2.4 (0.7)** | 0.7 (0.9)  **1.6 (0.7)** | 1.2 (1.3)  **0.6 (1.3)** | 0.5 (0.6)  **1.0 (0)** | 0.8 (0.9)  **1.2 (1.0)** | 1.6 (1.1)  **1.9 (1.3)** |
| Hedonic quality | 2.0 (0.8)  **2.2 (1.1)** | 2.0 (0.6)  **2.4 (0.6)** | 2.0 (0.9)  **1.9 (1.7)** | 2.0 (0.8)  **2.1 (1.2)** | 2.1 (0.8)  **2.4 (0.8)** | | | | 1.3 (1.1)  **1.5 (0.8)** | | | | 1.9 (0.9)  **2.1 (1.0)** |
| Overall | 2.0 (0.7)  **2.2 (0.7)** | 1.9 (0.6)  **2.5 (0.5)** | 1.9 (1.0)  **1.7 (1.7)** | 1.9 (0.8)  **2.1 (1.2)** | 2.0 (0.8)  **2.3 (0.7)** | | | | 1.4 (0.6)  **1.4 (0.6)** | | | | 1.8 (0.8)  **2.0 (0.9)** |

Abbreviations: UEQ-S, short version of the User Experience Questionnaire; NM, neuromuscular; OB, obesity; CP, cerebral palsy.

Table S2. Scores obtained on UMUX items for all participants; in children, parents and therapists; and in each subcategory. In each row, the first row of scores corresponds to results with non-immersive virtual reality, and the second row in bold to results with immersive virtual reality. Each item is a 7-point Likert scale (from 1 “strongly disagree” to 7 “strongly agree”). Data are expressed as means (standard deviations).

| UMUX items | Children | | | | Parents | | | | Therapists | | | | Overall  (N=74) |
| --- | --- | --- | --- | --- | --- | --- | --- | --- | --- | --- | --- | --- | --- |
|  | NM  (n=8) | OB  (n=9) | CP  (n=11) | Overall  (n=28) | NM  (n=8) | OB  (n=9) | CP  (n=11) | Overall  (n=28) | NM  (n=8) | OB  (n=9) | CP  (n=11) | Overall  (n=28) |  |
| This system’s capabilities meet my requirements. | 6,0 (0,9)  **6,3 (1,0)** | 5,9 (1,1)  **6,4 (0,7)** | 5,4 (1,9)  **5,0 (2,4)** | 5,7 (1,4)  **5,8 (1,7)** | 5,9 (1,0)  **5,9 (1,2)** | 5,9 (1,5)  **6,4 (0,9)** | 5,5 (0,7)  **5,5 (1,4)** | 5,7 (1,1)  **5,9 (1,3)** | 5,3 (1,0)  **5,0 (0,8)** | 5,0 (0,7)  **4,0 (1,4)** | 5,8 (0,5)  **5,3 (1,5)** | 5,3 (0,8)  **4,8 (1,2)** | 5,6 (1,2)  **5,6 (1,5)** |
| Using this system is a frustrating experience. | 1,3 (0,5)  **1,3 (0,5)** | 1,1 (0,3)  **1,1 (0,3)** | 2,4 (2,2)  **2,0 (2,2)** | 1,6 (1,5)  **1,5 (1,5)** | 1,4 (0,5)  **2,1 (1,9)** | 1,7 (0,7)  **1,9 (1,5)** | 1,9 (1,4)  **1,6 (1,2)** | 1,7 (1,0)  **1,9 (1,5)** | 2,8 (1,2)  **3,6 (1,4)** | 2,8 (1,6)  **2,2 (1,6)** | 2,0 (0)  **2,0 (1,4)** | 2,6 (1,2)  **2,8 (1,6)** | 1,9 (1,3)  **1,9 (1,6)** |
| This system is easy to use. | 5,8 (1,6)  **6,4 (0,7)** | 6,1 (0,9)  **6,6 (0,5)** | 5,8 (1,6)  **5,7 (1,7)** | 5,9 (1,4)  **6,2 (1,2)** | 6,0 (1,1)  **6,4 (0,5)** | 6,1 (1,4)  **6,3 (0,7)** | 6,0 (0,9)  **5,9 (0,7)** | 6,0 (1,1)  **6,2 (0,7)** | 5,9 (0,6)  **5,4 (0,9)** | 5,8 (0,8)  **5,8 (0,8)** | 5,0 (1,2)  **5,3 (0,5)** | 5,7 (0,8)  **5,5 (0,8)** | 5,9 (1,1)  **6,0 (1,0)** |
| I have to spend too much time correcting things with this system. | 2,0 (1,1)  **3,1 (1,6)** | 2,2 (1,7)  **2,1 (1,7)** | 2,1 (1,1)  **2,7 (1,8)** | 2,1 (1,3)  **2,6 (1,7)** | 2,1 (1,1)  **2,5 (1,4)** | 2,7 (2,0)  **1,9 (0,9)** | 2,5 (1,6)  **3,0 (1,9)** | 2,4 (1,6)  **2,5 (1,6)** | 3,2 (1,1)  **3,5 (1,9)** | 2,0 (0,7)  **2,4 (0,9)** | 3,0 (1,2)  **1,8 (1,5)** | 2,8 (1,1)  **2,8 (1,6)** | 2,4 (1,4)  **2,6 (1,6)** |

Abbreviations: UMUX, Usability Metric for User Experience; NM, neuromuscular; OB, obesity; CP, cerebral palsy.
